# Analysis of Bovine Kappa-Casein Glycomacropeptide by Liquid Chromatography–Tandem Mass Spectrometry

**DOI:** 10.3390/foods10092028

**Published:** 2021-08-28

**Authors:** Yunyao Qu, Bum-Jin Kim, Jeewon Koh, David C. Dallas

**Affiliations:** 1 Department of Food Science & Technology, Oregon State University, Corvallis, OR 97331, USA; yunyao.qu@oregonstate.edu; 2 Nutrition Program, School of Biological and Population Health Sciences, College of Public Health and Human Sciences, Oregon State University, Corvallis, OR 97331, USA; bumjin.kim@oregonstate.edu (B.-J.K.); kohje@oregonstate.edu (J.K.)

**Keywords:** LC–MS/MS, bovine casein, glycomacropeptide, top-down approach

## Abstract

Caseinomacropeptide (CMP) is released from bovine kappa-casein after rennet treatment and is one of the major peptides in whey protein isolate. CMP has in vitro anti-inflammatory and antibacterial activities. CMP has two major amino acid sequences with different modifications, including glycosylation, phosphorylation and oxidation. However, no previous work has provided a comprehensive profile of intact CMP. Full characterization of CMP composition and structure is essential to understand the bioactivity of CMP. In this study, we developed a top-down glycopeptidomics-based analytical method to profile CMP and CMP-derived peptides using Orbitrap mass spectrometry combined with nano-liquid chromatography with electron-transfer/higher-energy collision dissociation. The liquid chromatography–tandem mass spectrometry (LC–MS/MS) spectra of CMPs were annotated to confirm peptide sequence, glycan composition and other post-translational modifications using automatic data processing. Fifty-one intact CMPs and 159 CMP-derived peptides were identified in four samples (one CMP standard, two commercial CMP products and one whey protein isolate). Overall, this novel approach provides comprehensive characterization of CMP and CMP-derived peptides and glycopeptides, and it can be applied in future studies of product quality, digestive survival and bioactivity.

## 1. Introduction

Caseinomacropeptide (CMP) is a 64-amino-acid C-terminal fragment of bovine kappa-casein that is released after rennet (chymosin) treatment. It is the third most abundant protein/peptide in cheese whey after β-lactoglobulin and α-lactalbumin and accounts for 20–25% of whey protein [1].

CMP has numerous in vitro bioactivities, including neutralization of enterotoxin [2], inhibition of bacterial and viral adhesion to Caco-2 cells [3], promotion of bifidobacterial growth [4] and modulation of the immune system response [5]. Because of these bioactivities and its relative abundance in dairy products, researchers and the dairy industry are greatly interested in developing CMP-based functional foods. 

The bioactivity of CMP can vary with differing structure [6]. CMP is not a single structure but rather a complex of multiple forms varying in protein sequence and post-translational modifications (PTM), such as glycosylation, phosphorylation and oxidation. Genetic variants of kappa-casein lead to various peptide sequence variants of CMP. The most common variants of CMP specifically are the A and B variants, which differ by two amino acids (Asp^169^/Thr^157^ in variant A and Ala^169^/Ile^157^ in variant B) [7]. *O*-linked glycosylation (glycans linked to the oxygen atom of amino acids Thr or Ser) is the most common PTM in CMP. The degree of glycosylation of CMP is highly variable due to the different origins (breeds, lactation time and genetic variants) of milk sources and processing techniques used [8,9,10]. The fraction of CMP with glycosylation is herein referred to as glycomacropeptide (gCMP), whereas CMP without glycosylation is referred to as aglycosylated CMP (aCMP). The CMP sequence can have multiple *O*-glycosylation sites [1]. No *N*-linked glycans (glycans linked to the nitrogen atom of Asn of the peptide) have been demonstrated in gCMP [1]. Three compositions (four structures) of *O*-glycans have been identified in bovine kappa-casein-derived gCMP by high-performance liquid chromatography after *O*-glycan release by the alkaline borohydride reaction. The identified glycans include disaccharides (*N*-acetyl galactosamine and galactose, GalNAcGal), trisaccharides (*N*-acetyl galactosamine, galactose and *N*-acetyl neuraminic acid, GalNAcGalNeuAc, linear and branched) and tetrasaccharides (*N*-acetyl galactosamine, galactose and two *N*-acetyl neuraminic acids, GalNAc_1_Gal_1_NeuAc_2_), with relative amounts of 6.3%, 36.9% and 56.0%, respectively [11]. The majority of phosphorylation of CMP can occur at Ser^149^ and Ser^127^, revealed by analysis of digested caseinomacropeptide via MALDI-PSD-MS [12]. A minor tri-phosphorylated form of CMP has also been found at Thr^145^ [7,13]. The only methionine of CMP can be oxidized to methionine sulfoxide [8]. 

In the past, the bottom-up approach was mainly used for the analysis of CMP. In the bottom-up approach, samples are hydrolyzed with enzymes to create smaller glycopeptides/peptides followed by liquid chromatography (LC) with mass spectrometry (MS). This bottom-up method allowed characterization of glycan composition, glycosylation site and the site occupancy of the glycans in gCMPs. For instance, Hua et al. [14] analyzed the site-specific glycosylation of kappa-casein (which includes the sequence of CMP) after nonspecific protease digestion with pronase E via nano LC–MS/MS. The investigators reported the sites and the relative abundance of each site of *O*-glycosylation within the known sequence of gCMP as Thr^154^ (41%), Thr^163^ (29%), Thr^152^ (14%), Thr^142^ (7%) and Thr^157^ (0.1%). Four glycans, GalNAcGal, GalNAcGalNeuAc (linear and branched) and GalNAc_1_Gal_1_NeuAc_2_, were found. Holland et al. [15] performed in-gel pepsin digestion of kappa-casein and analysis using LC–MS/MS and found that the glycosylation site of the monoglycosylated form of gCMP was at Thr^152^, the glycosylation sites of the diglycosylated gCMP were at Thr^152^ and Thr^163^, and the glycosylation sites of the triglycosylated gCMP were at Thr^152^, Thr^154^ and Thr^163^. The protease digestion-based bottom-up approach allows the site-specific profiling of *O*-glycosylation in gCMP, but this approach loses key information, including how many *O*-glycans are simultaneously attached to a single gCMP, the extent to which other CMP modifications (phosphorylation and oxidation) are co-present with *O*-glycans within a single gCMP and the extent to which intact (64 amino acid length) gCMP and non-intact gCMP fragments are present in a sample [16]. 

The inability of the bottom-up approach to identify intact vs. fragment forms of CMP precludes its use in studies of the degree of survival of intact CMP and the release of fragments of CMP across digestion. Understanding the degree to which CMP is digested in humans is essential for determining the potential for CMP and its fragments to exert bioactivity within the human gut. Most in vitro studies of CMP bioactivity examine the intact form, ignoring the fact that CMP is exposed to a wide array of digestive proteases through the stomach and intestine, which could result in some degree of degradation. For future work examining the digestion of CMP in humans, it is essential to develop a novel analytical approach without the limitations of the bottom-up approach. Therefore, we herein created a top-down analytical approach, in which the sample would not be subjected to enzymatic digestion and be analyzed in its intact form by MS and MS/MS acquisitions. A top-down approach could overcome the information loss problem present in bottom-up analysis and provide identification and relative quantification of the intact gCMP and aCMP structures, including the number of glycosylations, co-present PTM and whether fragments of gCMP and aCMP are present in a sample. Beyond the application for examining CMP digestion, a top-down approach could be beneficial for monitoring CMP-containing product quality and consistency. For example, differing processing steps or quality of the original milk could affect the extent to which CMP is intact in the product and its degree and type of modification. Indeed, heat processing applied to milk or whey prior to CMP isolation is known to alter the amount of CMP glycosylation; more severe heating results in CMP with less glycosylation [17]. 

However, there was no currently available analytical method to determine the intact forms of all the structural variants of CMP in dairy products. Therefore, there was a need to develop methods to comprehensively characterize CMP structures. The full characterization of CMP would include determination of peptide sequences; identification of PTMs such as glycosylation, phosphorylation and oxidation; and determination of PTM sites on the peptide. The objective of this study was to comprehensively profile CMP and CMP-derived structures in commercial CMP powder and whey protein isolate (WPI) by a top-down approach with nano-LC/Orbitrap MS/MS. MS/MS fragmentation is required to determine CMP structure. Herein, we chose to apply electron-transfer/higher-energy collision dissociation (EThcD) fragmentation, a method that combines electron transfer dissociation (ETD) and higher-energy collisional dissociation (HCD). Compared with ETD or HCD alone, EThcD fragmentation allows a higher proportion of both peptide backbone and glycosidic bond fragmentations, enabling more confident *O*-glycopeptide characterization [18].

## 2. Materials and Methods

### 2.1. Sample Preparation

A commercially available CMP standard (CMP STD, caseinoglycopeptide from bovine casein, Sigma Aldrich, MO, USA), two CMP powders (CMP powder 1 and CMP powder 2, company information is restricted) provided by two dairy companies and one commercial WPI (Provon 290, Glanbia Nutritionals, Twin Falls, ID, USA) were obtained to analyze intact CMPs. Ten milligrams of each sample were completely dissolved in 10 mL of nanopure water (Barnstead, 18.2 MΩ) at room temperature and used in subsequent MS analysis without clean-up procedures. CMP powder 1 was composed of 95.9 % protein with approximately 95% CMP purity. CMP powder 2 was composed of 80% protein with approximately 95% CMP purity. WPI was composed of 90% protein.

### 2.2. LC/MS and MS/MS Analysis

Samples were analyzed using a Waters nanoACQUITY UPLC (Waters, Milford, MA, USA) with an Orbitrap Fusion™ Lumos™ Tribrid™ MS (Thermo Scientific, Waltham, MA, USA). Two microliters of the sample were diluted with 18 μL of nanopure water. One microliter of the diluted sample was loaded onto a C18 nanoACQUITY UPLC trap column (Waters, Milford, MA, USA, 180 μm × 20 mm, 5-μm bead) for enrichment and desalting, and separated by an Acquity UPLC Peptide BEH C18 column (Waters, Milford, MA, USA, 100 μm × 100 mm, 1.7 μm bead) for 30 min. All samples were separated at a flow rate of 0.5 µL/min with a gradient elution using solvent A (100% nanopure water with 0.1% formic acid) and solvent B (100% acetonitrile (Fisher Scientific, Waltham, MA, USA) with 0.1% formic acid (EMD Millipore, Billerica, MA, USA)): 3% to 5% B, 0 min to 5 min; 5% to 28.5% B, 5 min to 18.5 min; 28.5% to 100% B, 18.5 min to 22 min; 100% B, 22 min to 24.5 min; 100% to 3% B, 24.5 min to 25 min. Finally, the column was re-equilibrated with 97% A for 5 min.

Full-scan MS spectra were acquired in positive ionization mode over an *m*/*z* range of 200–2000 with a resolution of 60,000. The automatic gain control target was set to 4.0 × 10^5^, with a maximum injection time of 50 ms. The MS cycle time was set to 3 s. Following an MS scan, precursor compounds were automatically selected for MS/MS analysis by the acquisition software based on the following criteria: ion-intensity threshold 5.0 × 10^4^, charge state 2–8 and exclusion time 60 s. Selected precursor ions were fragmented using EThcD. The ETD reaction times were set depending on charge state (2+, 130 ms; 3+, 70 ms; 4+, 50 ms; 5+, 40 ms; 6+ to 8+, 20 ms) based on our previously described method [19], and supplemental higher energy collision dissociation activation was performed with 25% of collision energy. All MS/MS spectra were acquired in the positive ion mode over an *m*/*z* range of 300–2000 by the Orbitrap at resolution of 30,000. The automatic gain control target was set to 5.0 × 10^4^.

### 2.3. Data Analysis

**Figure 1:**
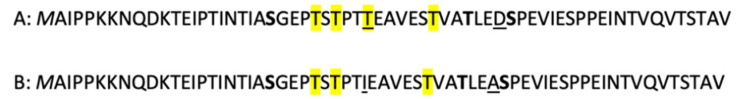
Sequences of A and B genetic variants of CMP with possible modification sites from literature search. Underlined amino acids are those that differ between the A and B sequences. Highlighted amino acids are those previously identified as *O*-glycosylation sites. Bolded amino acids are those with previously identified phosphorylation ref. Italicized amino acids are those with possible oxidation.

Raw files were analyzed by database searching in Byonic v.3.8.13 (Protein Matrix, Inc., New York, NY, USA) using genetic variant CMP sequences A and B (Asp^169^/Thr^157^ in variant A and Ala^169^/Ile^157^ in variant B) as the protein database (Figure 1). The precursor mass tolerance was set to 10 ppm, with fragment mass tolerance of 20 ppm. Potential modifications allowed included phosphorylation of Ser and Thr, oxidation of Met and *O*-linked glycosylation of Ser and Thr. The possible *O*-linked glycan library was based on those most common and abundant for gCMP, including GalNAcGal, GalNAcGalNeuAc and GalNAc_1_Gal_1_NeuAc_2_ [11]. Outputs of proteins were filtered at a 1% false discovery rate, as calculated by Byonic. Outputs of peptides were automatically filtered within 0–5% false discovery rate based on the protein filtering outputs (automatic score cut-off). As an additional filter to ensure quality data, glycopeptide–spectra matches with PEP 2D (protein-aware posterior error probability) < 0.01 (is equal to |Log Prob| > 2) and score > 100 were retained for the reported results. These filtering conditions were based on previous studies [20,21,22]. Multiple charge states of CMPs and CMP fragments were grouped into a single peptide for counting the number of peptides in a sample. The area under the curve of the eluted peak based on ion intensity was measured to calculate the abundance. The total abundance of peptides reported in the results was a sum of individual CMP and CMP fragment abundances.

## 3. Results and Discussion

Our approach of directly injecting CMP and WPI powders without any protease digestion or clean-up into the C18-nano LC system and analysis using EThcD-based MS/MS acquisition with an automated glycopeptide search and assignment tool allowed the identification of intact CMPs and CMP fragments with (1) peptide sequences derived from CMP A and B variants, (2) single or multiple *O*-glycans and (3) multiple PTMs.

### 3.1. Confirmation of Non-Glycosylated CMP (aCMP) Sequence Variants

An example tandem MS spectrum of the intact aCMP with sequence A (*m*/*z* 1341.661, *z* = 5) demonstrates comprehensive coverage across the intact CMP sequence, including fragment ions b2-b43, c2-c31 and y2-c18 (Figure S1a). The A variant was confirmed based on the c31 ion (*m*/*z* 1640.852, *z* = 2), which included Thr^157^ and the b43 ion (*m*/*z* 1504.095, *z* = 3), which included Asp^169^ (the two amino acids that differentiate between the A and B CMP genetic variants). Likewise, for aCMP with sequence B (*m*/*z* 1335.294, *z* = 5), ion coverage was good, including b2-b43, c2-c31 and y2-y17, and the B variant was confirmed based on the c31 ion (*m*/*z* 1646.865, *z* = 2), which included Ile^157^ and the b43 ion (*m*/*z* 1493.449, *z* = 3), which included Ala^169^ (Figure S1b).

### 3.2. Confirmation of gCMP Structures with Single or Multiple O-glycans

**Figure 2:**
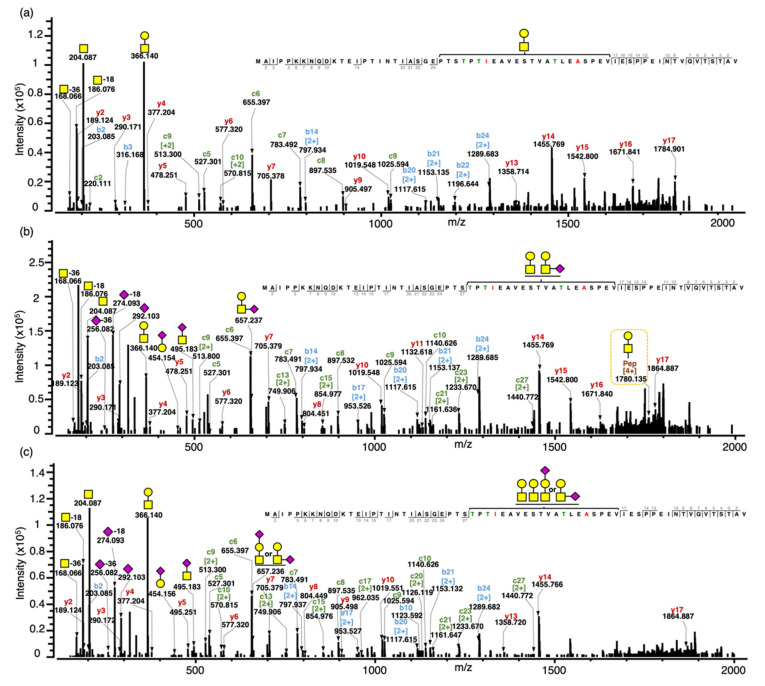
Annotated tandem MS spectra for intact gCMP with sequence B containing one, two and three *O*-glycans. (**a**) Tandem MS spectrum for the intact gCMP with sequence B containing one *O*-glycan (GalNAcGal) and one phosphate, observed *m*/*z* 1424.304 [M+5H]^5+^, scan time = 21.96 min found in the CMP powder 1 sample. (**b**) Tandem MS spectra for the intact gCMP with sequence B containing two *O*-glycans (GalNAcGal and GalNAcGalNeuAc) and one phosphate, observed *m*/*z* 1555.544 [M+5H]^5+^, scan time = 21.84 min found in the CMP standard sample. (**c**) Tandem MS spectra for the intact gCMP with sequence B containing three *O*-glycans (GalNAcGal, GalNAcGal and GalNAcGalNeuAc) and one phosphate, observed *m*/*z* 1628.582 [M+5H]^5+^, scan time = 21.25 min found in the CMP powder 2 sample. Glycan symbols: yellow square, *N*-acetyl galactosamine; yellow circle, galactose; and purple diamond, *N*-acetyl neuraminic acid.

Select glycopeptide–spectral matches determined by Byonic were confirmed via manual inspection and annotation. As an example of the confirmation of the peptide sequence and glycan composition, three MS/MS spectra from the same sequence (CMP B) containing one, two and three *O*-glycans were selected (Figure 2). The interpretation of tandem MS spectra for the intact gCMP with sequence B containing one *O*-glycan (GalNAcGal) and one phosphate (observed *m*/*z* 1424.306, *z* = 5), two *O*-glycans (GalNAcGal and branched GalNAcGalNeuAc) and one phosphate (observed *m*/*z* 1555.544, *z* = 5), three *O*-glycans (two GalNAcGal, linear or branched GalNAcGalNeuAc) and one phosphate (observed *m*/*z* 1628.582, *z* = 5) are shown in Figure 2a–c, respectively.

The glycopeptide compositions were confirmed based on oxonium ions, peptide backbone fragments and intact mass. The oxonium ions were used to confirm the glycan moiety. Oxonium ions from *O*-glycan fragmentation of the glycopeptide were mostly abundant at the lower mass range in all of the tandem MS spectra. For example, these glycopeptide precursor ions both generated representative oxonium ions—*m*/*z* 168 (GalNAc-36 (2OH & 2H), 186 (GalNAc-18 (OH & H)), 204 (GalNAc) and 366 (GalNAcGal)—of complex type glycans and the sialylated glycopeptide additionally included the diagnostic fragment ions of sialic acid such as *m*/*z* 274 (NeuAc-18 (OH & H)), 292 (NeuAc), 454 (GalNeuAc), 495 (GalNAcNeuAc), 657 (GalNAcGalNeuAc) and 948 (GalNAc_1_Gal_1_NeuAc_2_) (Figure S2). Holland et al. [13] found the same representative oxonium ions—*m*/*z* 204 (GalNAc), 292 (NeuAc), 366 (GalNAcGal), 454 (GalNeuAc), 495 (GalNAcNeuAc) and 657 (GalNAcGalNeuAc)—from a doubly charged parent ion at *m*/*z* 980 (IASGEPTSTPTIE- GMP B Ile^125^ to Gle^137^ carrying a GalNAcGalNeuAc) from the peptic digest of kappa-casein subjected to MS/MS. The peptide backbones of these gCMP were confirmed by the presence of singly and doubly charged c-ions (c2–27), b-ions (b2–24) and y-ions (y2–17) on the three tandem MS spectra. Intact peptides with partially fragmented glycans were observed in some spectra, providing added confirmation that the ascribed glycopeptide composition was accurate—for example, the quadruply charged intact peptide sequence with GalNAcGal at *m*/*z* 1780.135 in Figure 2b.

Both linear and branched structural isomers of the *O*-glycan composition, GalNAcGalNeuAc, were found in a single tandem MS spectrum (Figure 2c). The oxonium ions, GalNeuAc (*m*/*z* 454) and GalNAcNeuAc (*m*/*z* 495), fragmented from GalNAcGalNeuAc are diagnostic for the linear structure with NeuAc attached on the outer Gal and the branched structure with NeuAc attached on the core GalNAc, respectively. Therefore, the MS spectra indicate that these isomers co-eluted and were analyzed at the same time, allowing two different intact forms to be observed in the same spectra. Both structures were identified by the current method; however, glycopeptides with different glycan isomers could not be separated by retention time with a C18 column. Previous works have demonstrated that porous graphitized carbon columns could be used to separate released *O*-glycan isomers [23] and pronase-digested glycopeptides [24]. Future analysis of CMP could try replacing the C18 LC column with a porous graphitized carbon LC column to increase the separation of the *O*-glycan isomers. However, the larger the peptide moiety, the less is the impact of the glycan on retention in graphitized carbon columns [25]; thus, glycan isomer separation for intact gCMP is unlikely. 

Our current method cannot determine the exact glycan sites within the intact CMP sequence, but the three tandem MS spectra were able to limit the region of possible *O*-glycan sites. In the three tandem MS spectra, only peptide sequences 2–27 AA and 2–17 AA on the N- and C-terminal, respectively, were observed. Neither glycan nor peptide fragment ions were observed between Thr^154^ and Val^171^. As no fragments with both peptide and glycan were present on the N- and C-terminal segments observed, we know that the glycan is present on a site between Thr^154^ and Val^171^. This finding matches the previously identified, most common glycosylation sites for gCMP, including Thr^154^, Thr^163^ and Thr^152^. The lack of fragmentation between Thr^154^ and Val^171^ precluded our ability to determine the exact glycosylation site. Interestingly, though coverage was missing between Thr^154^ and Val^171^ for gCMP, the peptide fragmentation for intact aCMP was comprehensive, including fragments between Thr^154^ and Val^171^. This lower fragmentation coverage for gCMP may indicate that the glycosylation structure alters the peptide fragmentation pattern [26]. As HCD at a higher energy can increase the coverage of fragment ions across a peptide sequence [18], it is possible that increasing the HCD energy in future work will provide these interior peptide fragmentations to enable glycosite determination. Moreover, as careful calibration of ETD reaction times can increase the coverage rate [27], future ETD optimization may enable increased intact CMP identification. However, as non-glycosylated forms of intact CMP demonstrated comprehensive fragment ion coverage (Figure S1), the presence of glycosylation directly inhibited the formation of the fragment ions that would enable glycosite determination (between AA 28 and 40). Therefore, increasing HCD energy and ETD reaction time may not increase fragmentation between these sites due to properties inherent to the large glycopeptide structure. We were, however, able to determine the glycosylation site of some gCMP fragments. For example, MAIPPKKNQDKTEIPTINTIASGEPTSTPTTEAVESTVATLED + GalNAc_1_Gal_1_NeuAc_2_ (*m*/*z* 1369.159, *z* = 4) had a glycosylation site at Thr^154^ (Figure S3). However, for the most part, exact glycosylation sites could not be determined for gCMP fragments, likely due to their relatively long AA sequences (average length AA 57 ± 7).

### 3.3. Confirmation of gCMP with Multiple Modifications (Phosphorylation and Oxidation)

**Figure 3:**
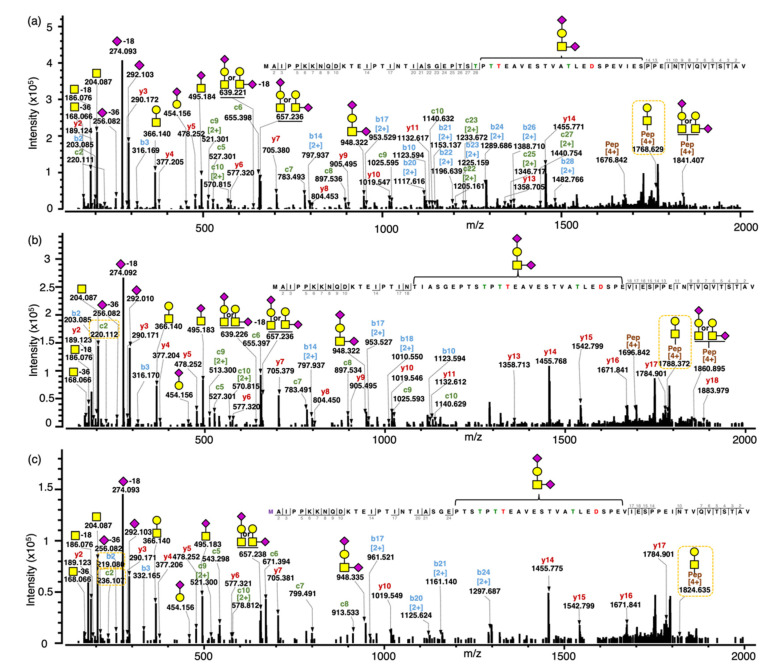
Annotated tandem MS spectra of intact CMPs with no, single or multiple modifications. (**a**) Tandem MS spectra for the intact gCMP with sequence CMP A containing one *O*-glycan (GalNAc_1_Gal_1_NeuAc_2_) and one phosphate, observed *m*/*z* 1547.149 [M+5H]^5+^, scan time = 21.71 min found in the WPI sample; (**b**) Tandem MS spectra for the intact gCMP with sequence CMP A containing one *O*-glycan (GalNAc_1_Gal_1_NeuAc_2_), one phosphate and one oxidation, observed *m*/*z* 1550.348 [M+5H]^5+^, scan time = 21.81 min found in the CMP powder 1 sample; (**c**) Tandem MS spectra for the intact gCMP with sequence CMP A containing one *O*-glycan (GalNAc_1_Gal_1_NeuAc_2_), observed *m*/*z* 1531.151 [M+5H]^5+^, scan time = 21.98 min found in the CMP powder 1 sample. Glycan symbols: yellow square, *N*-acetyl galactosamine; yellow circle, galactose; and purple diamond, *N*-acetyl neuraminic acid.

To confirm the composition/structure of intact gCMPs with phosphorylation and oxidation, two select MS/MS spectra of gCMPs from the same sequence (CMP A) containing (1) one *O*-glycan, GalNAc_1_Gal_1_NeuAc_2_ and one phosphorylation and (2) one *O*-glycan, GalNAc_1_Gal_1_NeuAc_2_, one phosphorylation and one oxidation were annotated (Figure 3a,b) and compared with an MS/MS spectrum of gCMP with only GalNAc_1_Gal_1_NeuAc_2_ (Figure 3c).

In Figure 3c, the intact gCMP with sequence CMP A containing one *O*-glycan GalNAc_1_Gal_1_NeuAc_2_, a quadruply charged fragment ion for GalNAcGal + intact peptide sequence, was observed at *m*/*z* 1768.384. In Figure 3a, the same gCMP sequence with one phosphorylation showed the quadruply charged fragment ion for GalNAcGal + intact peptide sequence at *m*/*z* 1788.374, which is 19.990 *m*/*z* (80 Da in the neutral mass) higher due to the phosphorylation. Tablo et al. [10] found that CMP can be phosphorylated at Ser^22^ and Ser^44^ of CMP (Ser^148^ and Ser^170^, respectively, of kappa-casein, counting the signaling sequence) by analyzing digested caseinomacropeptide by MALDI-PSD-MS. On the digested CMPs, the authors were able to find the exact phosphorylation sites, confirmed with specific fragment ions with +80 Da. However, in our data, though we could determine the number of phosphorylations present on the intact gCMP, we could not determine the site of phosphorylation, as fragments containing Ser^22^ and Ser^44^ of CMP were not identified (b- and c-ions covered AA 2–18 from the N-terminal and y-ions covered AA 2–18 from the C-terminal). The exact site of phosphorylation was, however, possible to obtain for the smaller CMP fragments. For example, pep DSPEVIESPPEINTVQVTSTAV (*m*/*z* 2276.082, *z* = 2) had a phosphorylation site at Ser^170^ (Figure S4). The previously identified sites of phosphorylation (Ser^148^ and Ser^170^) were confirmed in the smaller CMP fragments.

The fragmentation ions of the peptide chain with oxidation from the N-terminal were 16 Da higher than those of the peptide chain without oxidation; for example, all observed b ions (b2–b18) and c ions (c2–c10) were 16 Da higher in the tandem MS spectrum of CMP with oxidation (Figure 3c) than in the CMP without oxidation (Figure 3b). The oxidation can happen only on the methionine, which is the first amino acid from the N-terminal of CMP.

Overall, this current method successfully identified aCMP with sequence variants, gCMP with specific *O*-linked glycosylations and other modifications, including phosphorylation and oxidation.

### 3.4. Overall Findings of Peptides in Samples

Our top-down LC–MS/MS approach enabled the identification of intact aCMP and gCMP and fragments of aCMP and gCMP. This approach allowed us to distinguish between CMP sequence variants (CMP A vs. B) and identify gCMP with single and multiple *O*-glycans and multiple PTM (phosphorylation and/or oxidation). Previous studies found three kinds of *O*-linked glycosylations on CMP—GalNAcGal (disaccharide), GalNAcGalNeuAc (trisaccharide) and GalNAc_1_Gal_1_NeuAc_2_ (tetrasaccharide) [9]. All of these previously identified *O*-glycans were present in the intact gCMP and fragment gCMP in our data (disaccharide-gCMP in Figure 2, trisaccharide-gCMP in Figure S5 and tetrasaccharide-gCMP in Figure 3).

We applied the method developed herein to analyze the CMP profile of four commercially available CMP products (CMP STD, CMP powder 1, CMP powder 2 and WPI). Across all analyzed samples, a total of 211 CMPs and CMP fragments were found, including 51 intact CMPs (both gCMP and aCMP) (listed in Table S1) and 159 CMP fragments (both gCMP and aCMP fragments) (listed in Table S2). To be counted as a unique composition, a peptide had to have a different CMP variant (CMP A and B), different composition of *O*-glycans (GalNAcGal, GalNAcGalNeuAc and GalNAc_1_Gal_1_NeuAc_2_) or different combinations of PTMs (0-3 *O*-glycosylation, 0-2 phosphorylation and/or 0-1 oxidation).

The number of intact CMP and CMP fragments in each sample (CMP STD, CMP powder 1, CMP powder 2 and WPI) was similar. In the CMP STD, CMP powder 1, CMP powder 2 and WPI, we found 48, 49, 51 and 50 variants of intact CMPs, respectively, and 149, 154, 150 and 153 CMP fragments, respectively (Table 1).

CMP powder 1, CMP powder 2 and WPI showed a similar trend in terms of the relative abundance of gCMP (18.0–6.7%), aCMP (39.4–53.9%), gCMP fragments (23.5–30.2%) and aCMP fragments (1.4–3.7%) (Table 2). The CMP STD had a higher relative abundance of gCMP (53.8%) and lower abundance of aCMP (10.6%) than the other samples (Table 2). This finding may reflect the different processing methods used for the isolation and purification of the CMP STD compared with the other samples. In all four samples, 64.4–5.0% of the observed peptides were in the intact form (gCMP and aCMP). The presence of a relatively large portion (25.0–35.6%) of fragment peptides suggests that the intact CMP sequence experiences degradation during processing and storage. If bacterial cultures are added to milk directly during cheesemaking, they could induce CMP proteolysis. Moreover, milk itself contains an array of other native proteases (e.g., plasmin, cathepsins, elastase) and native bacteria that may interact with the CMP sequence during processing [28].

**Figure 4:**
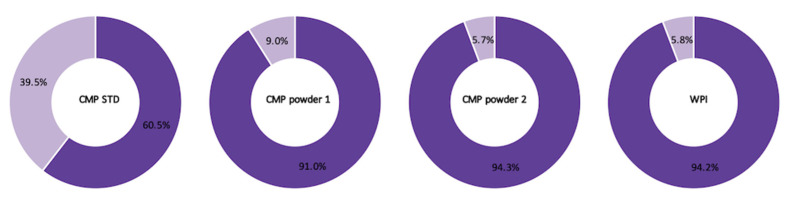
The relative abundances of intact CMP with CMP A (purple) and CMP B (light purple) sequence in the four samples (CMP STD, CMP powder 1, CMP powder 2 and WPI).

The average counts of intact CMP with A or B sequences in the four analyzed samples (CMP STD, CMP powder 1, CMP powder 2 and WPI) were approximately equal—there were, on average, 25 CMP A and 25 CMP B. CMP powder 1, CMP powder 2 and WPI showed a similar trend in terms of the relative abundance of intact gCMP with the A or B sequence—91.0–94.3% intact CMP with A sequence and 5.7–9.0% intact CMP with B sequence (Figure 4). The CMP standard, on the other hand, had 60.5% intact CMP with A sequence and 39.5% intact CMP with B sequence (Figure 4). This difference could be attributed to differing breeds and/or lactation stages of the cows providing the milk used to produce each CMP fraction [29,30].

Across the samples analyzed, the average counts of intact CMP containing one, two and three *O*-glycans were 18, 15 and 9, respectively (Table S3). CMP powder 1, CMP powder 2 and WPI showed a similar trend in terms of the relative abundance of intact gCMP: 78.8–80.7% intact gCMP with one *O*-glycan, 17.1–19.5% with two *O*-glycans and 1.5–4% with three *O*-glycans. The CMP standard also only had 3% gCMP with three *O*-glycans but more gCMP with two *O*-glycans (43.0%) and less gCMP with one *O*-glycan (53.7%) compared with the other three samples (Table 2).

### 3.5. Limitations of the Study

One limitation of our approach was that the combination of the C18 analytical column and the elution gradient did not allow separation of glycan structural isomers. This co-elution may limit the differentiation of isomeric gCMP structures. As mentioned, other liquid chromatography columns such as porous graphitized carbon can be investigated in future work to allow glycan isomer separation. However, as stated earlier, the long peptide chain of the gCMP in these samples may limit the capacity for separation based on the glycan component. 

Another limitation of our study was the lack of glycosite information, particularly for intact gCMP. EThcD fragmentation during the mass spectrometry analysis provided both oxonium and peptide moiety fragment ions in the tandem MS spectra (Figure 2 and Figure 3). This hybrid fragmentation method produced rich fragment ions derived from both the N- and C-terminals of the peptide moiety (b- and y-ions from HCD and c-ions from ETD), which allowed clear determination of the peptide sequence. However, this approach did not provide peptide fragmentation between the *O*-glycosylation sites, precluding exact glycosite determination. As mentioned, further optimization of the HCD and ETD parameters may enable improved fragmentation and glycosite determination. 

In our current study, we utilized the label-free quantitation method to provide the relative abundances by determining the precursor ion intensity based on the extracted ion chromatogram, which is the plot of intensity versus retention time of a certain *m*/*z* value [23]. Our results only show the relative abundances of intact CMP and fragmented CMP. Absolute quantitation in future work will be necessary to determine the exact amount of intact CMP and fragmented CMP.

## 4. Conclusions

Our study comprehensively profiled CMP and CMP-derived structures in commercial CMP and WPI powders using a top-down approach with C18-nano-LC/Orbitrap MS/MS. The Orbitrap mass spectrometer provides high dynamic range and high mass resolution, essential in identifying these CMP structures [31]. Our MS/MS results confirmed the intact and fragment CMP structures, including multiple *O*-glycosylations, phosphorylations and oxidation.

Previous studies used a bottom-up approach and were able to identify smaller, digested fragments (~10 amino acids) with a single glycosylation site [15]. Our top-down approach enabled the identification of intact CMP, which is much longer (64 amino acids in length), the composition of *O*-linked glycans and the number of glycans per peptide backbone as well as the compositions of fragment CMP already present in the analyzed dairy products. Data processing using software that interprets the LC/MS and MS/MS data allowed the automatic assignment of fragment ions to determine the glycan compositions and peptide sequences of the complex CMPs as well as CMP fragments. 

This top-down method for analyzing CMP can be applied to determine the extent to which intact CMP survives and CMP fragments are released across gastrointestinal digestion in humans. We currently lack information about the extent of survival of CMP across the human gut. Such research is essential to help to determine the biological relevance of CMP and CMP fragments within the gut. This approach may also be helpful in determining the purity of commercial CMP products and to monitor product quality, including the extent of off-target hydrolysis and glycosylation. This information could enable dairy processors to modify their processing techniques to produce certain forms of CMP that carry a higher degree of bioactivity. 

## Figures and Tables

**Table 1:** Count of aCMP, gCMP, fragment aCMP and fragment gCMP in four samples (CMP STD, CMP powder 1, CMP powder 2 and WPI).

	CMP STD	CMP Powder 1	CMP Powder 2	WPI
aCMP	8	8	8	8
gCMP	40	41	43	42
fragment aCMP	44	49	48	48
fragment gCMP	105	105	102	105

**Table 2:** The relative abundances of aCMP, gCMP, fragment aCMP and fragment gCMP in the four samples (CMP STD, CMP powder 1, CMP powder 2 and WPI). Abundances marked with * are the relative abundance of the 1 *O*-glycan, 2 *O*-glycans and 3 *O*-glycans within the intact gCMPs in the four samples.

	GMP STD	CMP Powder 1	CMP Powder 2	WPI
aCMP	10.6%	39.4%	53.9%	51.6%
gCMP	53.8%	26.7%	21.1%	18.0%
1 *O*-glycan	53.7% *	79.0% *	80.7% *	78.8% *
2 *O*-glycans	43.0% *	19.5% *	17.8% *	17.1% *
3 *O*-glycans	3.3% *	1.5% *	1.5% *	4.0% *
fragment aCMP	1.4%	3.7%	1.4%	2.9%
fragment gCMP	23.5%	30.2%	23.5%	27.5%

## Data Availability

The data presented in this study are available in the article and supplementary material here.

## Supplementary Materials

The following are available online at https://www.mdpi.com/article/10.3390/foods10092028/s1, Figure S1. MS/MS spectra for intact aCMP with sequence A and B. (a) Tandem MS spectra for the intact aCMP with sequence A, observed *m*/*z* 1341.661, [M+5H]^5+^, scan time = 21.90 min found in the CMP powder 2 sample. (b) Tandem MS spectra for the intact aCMP with sequence B, observed *m*/*z* 1335.294 [M+5H]^5+^, scan time = 22.23 min found in the CMP powder 2 sample. Figure S2. The representative oxonium ions identified from intact gCMPs and gCMP fragments (GalNAcGal, GalNAcGalNeuAc and GalNAc_1_Gal_1_NeuAc_2_). Glycan symbols: yellow square, *N*-acetyl galactosamine; yellow circle, galactose; and purple diamond, *N*-acetyl neuraminic acid. Figure S3. Annotated MS/MS spectra for gCMP fragment with sequence A one *O*-glycan. Tandem MS spectra for MAIPPKKNQDKTEIPTINTIASGEPTSTPTTEAVESTVATLED + GalNAc_1_Gal_1_NeuAc_2_ (*m*/*z* 1369.159, *z* = 4) had a glycosylation site at Thr^154^ in the CMP standard sample. Glycan symbols: yellow square, *N*-acetyl galactosamine; yellow circle, galactose; and purple diamond, *N*-acetyl neuraminic acid. Figure S4. Annotated MS/MS spectra for aCMP fragment with sequence A with phosphorylation. Tandem MS spectra for DSPEVIESPPEINTVQVTSTAV (*m*/*z* 2276.082, *z* = 2) had a phosphorylation site at Ser^170^ found in CMP powder 1 sample. Figure S5. Annotated MS/MS spectra for intact gCMP with sequence A containing trisaccharide *O*-glycan. Tandem MS spectra for the intact gCMP with sequence B containing one *O*-glycan (GalNAcGalNeuAc) and one phosphate, observed *m*/*z* 1488.924 [M+5H]^5+^, scan time = 21.68 min found in the CMP standard sample. Glycan symbols: yellow square, *N*-acetyl galactosamine; yellow circle, galactose; and purple diamond, *N*-acetyl neuraminic acid. Table S1. The 51 intact CMPs found in the four samples with peptide sequence, number of phosphorylations, number of oxidations, number of *O*-glycosylations, glycan structure, observed mass, calculated mass and error ppm. Table S2. The 159 CMP fragments found in the four samples with peptide sequence, number of phosphorylations, number of oxidations, number of *O*-glycosylations, glycan structure observed mass, calculated mass and error ppm. Table S3. The counts of intact gCMP containing 1, 2 and 3 *O*-glycans identified in the four samples (CMP STD, CMP powder 1, CMP powder 2 and WPI).
