# Supplementary material for: Analysis of Bovine Kappa-Casein Glycomacropeptide by Liquid Chromatography–Tandem Mass Spectrometry
Source: Foods. 2021 Aug 28;10(9):2028. doi: 10.3390/foods10092028 (PMC8469635; doi:10.3390/foods10092028)
Supplement: Supplementary file 1 [file foods-10-02028-s001.zip › Sup Figures and tables.pdf]

Analysis of Bovine Kappa-Casein Glycomacropeptide by Liquid Chromatography-Tandem Mass Spectrometry

Yunyao Qu [1,2], Bum Jin Kim [2], Jeewon Koh [2] and David C. Dallas [2,*]

[1]*Department of Food Science & Technology, Oregon State University, Corvallis, OR 97331*

[2]*Nutrition Program, School of Biological and Population Health Sciences, College of Public Health and Human Sciences, Oregon State University, Corvallis, OR 97331*

***** *To whom correspondence should be addressed: David C. Dallas, 118G Milam Hall, Corvallis, OR 97331. Tel.: 541-737- 1751.*

E-mail address dave.dallas@oregonstate.edu

Conflict of interest statement: The authors have declared no conflict of interest

***Corresponding author at** David C. Dallas; 118G Milam Hall, Corvallis, OR 97331; 541-737-1751; dave.dallas@oregonstate.edu

Table of Content

FIGURE S1. MS/MS SPECTRA FOR INTACT ACMP WITH SEQUENCE A AND B. A) TANDEM MS SPECTRA FOR THE INTACT ACMP WITH SEQUENCE A, OBSERVED M/Z 1341.661, [M+5H]5+, SCAN TIME = 21.90 MIN FOUND IN THE CMP POWDER 2 SAMPLE. B) TANDEM MS SPECTRA FOR THE INTACT ACMP WITH SEQUENCE B, OBSERVED M/Z 1335.294[M+5H]5+, SCAN TIME = 22.23 MIN FOUND IN THE CMP POWDER 2 SAMPLE....................................S-3

FIGURE S2. THE REPRESENTATIVE OXONIUM IONS IDENTIFIED FROM INTACT GCMPSAND GCMP FRAGMENTS (GALNACGAL, GALNACGALNEUAC AND GALNAC$_1$GAL$_1$NEUAC$_2$). GLYCAN SYMBOLS: YELLOW SQUARE, N-ACETYL GALACTOSAMINE; YELLOW CIRCLE, GALACTOSE; AND PURPLE DIAMOND, N-ACETYL NEURAMINIC ACID......... S-4

FIGURE S3. ANNOTATED MS/MS SPECTRA FOR GCMP FRAGMENT WITH SEQUENCE A ONE O-GLYCAN. TANDEM MS SPECTRA FOR MAIPPKKNQDKTEIPTINTIASGEPTSTPTTEAVESTVATLED + GALNAC1GAL1NEUAC2 (M/Z 1369.159, Z=4) HAD A GLYCOSYLATION SITE AT THR154 IN THE CMP STANDARD SAMPLE. GLYCAN SYMBOLS: YELLOW SQUARE, N-ACETYL GALACTOSAMINE; YELLOW CIRCLE, GALACTOSE; AND PURPLE DIAMOND, N-ACETYL NEURAMINIC ACID..S-5

FIGURE S4. ANNOTATED MS/MS SPECTRA FOR ACMP FRAGMENT WITH SEQUENCE A WITH PHOSPHORYLATION. TANDEM MS SPECTRA FOR DSPEVIESPPEINTVQVTSTAV (M/Z 2276.082, Z=2) HAD A PHOSPHORYLATION SITE AT SER170 FOUND IN CMP POWDER 1 SAMPLE...S-6

FIGURE S5. ANNOTATED MS/MS SPECTRA FOR INTACT GCMP WITH SEQUENCE A CONTAINING TRISACCHARIDE O-GLYCAN. TANDEM MS SPECTRA FOR THE INTACT GCMP WITH SEQUENCE B CONTAINING ONE O-GLYCAN (GALNACGALNEUAC) AND ONE PHOSPHATE, OBSERVED M/Z 1488.924 [M+5H]5+, SCAN TIME = 21.68 MIN FOUND IN THE CMP STANDARD SAMPLE. GLYCAN SYMBOLS: YELLOW SQUARE, N-ACETYL GALACTOSAMINE; YELLOW CIRCLE, GALACTOSE; AND PURPLE DIAMOND, N-ACETYL NEURAMINIC ACID..S-7

TABLE S1. THE 51 INTACT CMPS FOUND IN THE FOUR SAMPLES WITH PEPTIDE SEQUENCE, NUMBER OF PHOSPHORYLATIONS, NUMBER OF OXIDATIONS, NUMBER OF O-GLYCOSYLATIONS, GLYCAN STRUCTURE, OBSERVED MASS, CALCULATED MASS AND ERROR PPM...S-8

TABLE S2. THE 159 CMP FRAGMENTS FOUND IN THE FOUR SAMPLES WITH PEPTIDE SEQUENCE, NUMBER OF PHOSPHORYLATIONS, NUMBER OF OXIDATIONS, NUMBER OF O-GLYCOSYLATIONS, GLYCAN STRUCTURE OBSERVED MASS, CALCULATED MASS AND ERROR PPM...S-8

TABLE S3. THE COUNTS OF INTACT GCMP CONTAINING 1, 2 AND 3 O-GLYCANS IDENTIFIED IN THE FOUR SAMPLES (CMP STD, CMP POWDER 1, CMP POWDER 2 AND WPI)...S-8

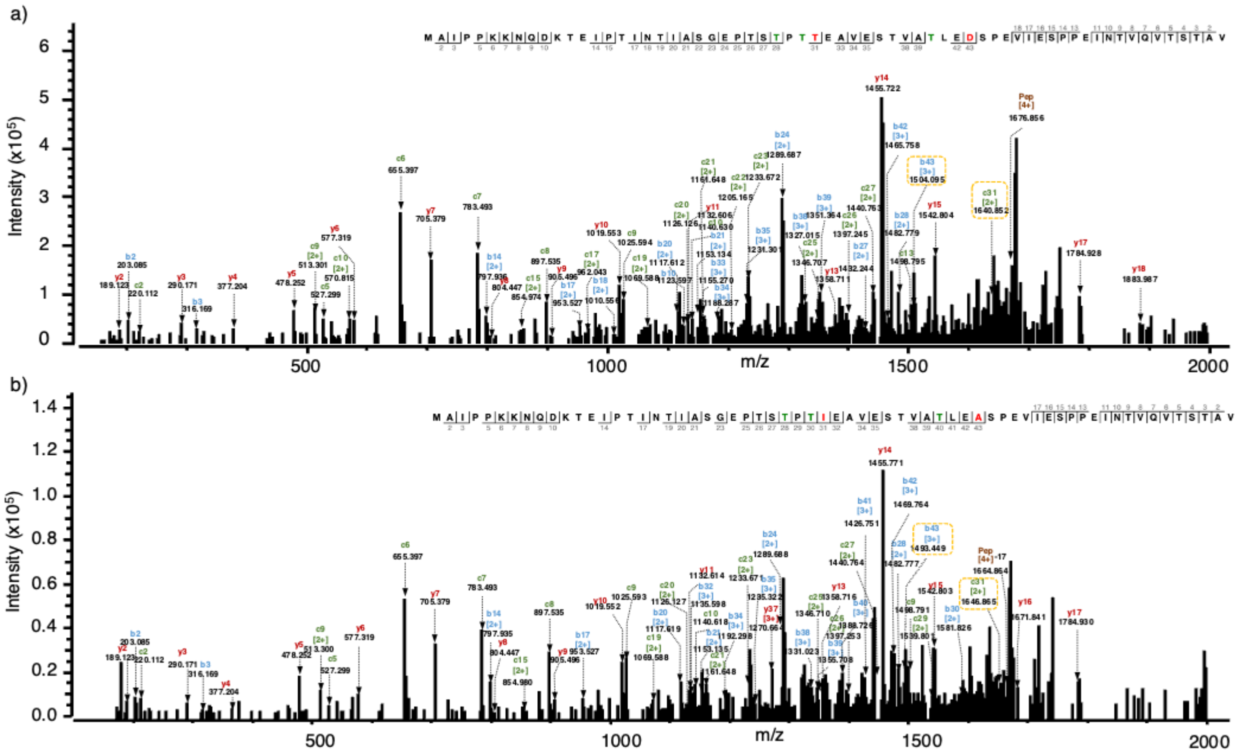

Figure S1. MS/MS spectra for intact aCMP with sequence A and B. a) Tandem MS spectra for the intact aCMP with sequence A, observed m/z 1341.661, [M+5H]5+, scan time = 21.90 min found in the CMP powder 2 sample. b) Tandem MS spectra for the intact aCMP with sequence B, observed m/z 1335.294[M+5H]5+, scan time = 22.23 min found in the CMP powder 2 sample.

m/z [M+H]$^+$	Composition	Structure
168.065	GalNAc-36	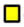 -2H & -2OH
186.076	GalNAc-18	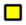 -H & -OH
204.087	GalNAc	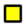
274.092	NeuAc-18	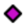 -H & -OH
292.103	NeuAc	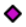
366.140	GalNAcGal	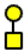
454.156	GalNeuAc	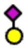
495.182	GalNAcNeuAc	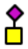
639.225	GalNAcGalNeuAc-18	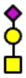 -H & -OH or 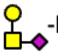 -H & -OH
657.235	GalNAcGalNeuAc	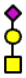 or 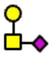
948.330	$Gal_1NAcGal_1NeuAc_2$	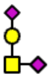

Figure S2. The representative oxonium ions identified from intact gCMPsand gCMP fragments (GalNAcGal, GalNAcGalNeuAc and $GalNAc_1Gal_1NeuAc_2$). Glycan symbols: yellow square, N-acetyl galactosamine; yellow circle, galactose; and purple diamond, N-acetyl neuraminic acid.

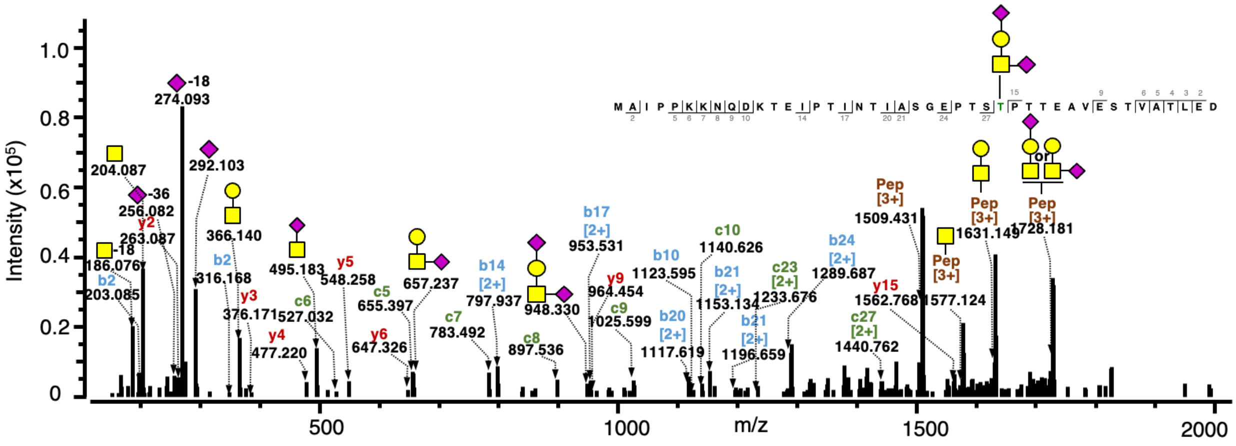

Figure S3. Annotated MS/MS spectra for gCMP fragment with sequence A one O-glycan. Tandem MS spectra for MAIPPKKNQDKTEIPTINTIASGEPTSTPTTEAVESTVATLED + GalNAc1Gal1NeuAc2 (m/z 1369.159, z=4) had a glycosylation site at Thr154 in the CMP standard sample. Glycan symbols: yellow square, N-acetyl galactosamine; yellow circle, galactose; and purple diamond, N-acetyl neuraminic acid.

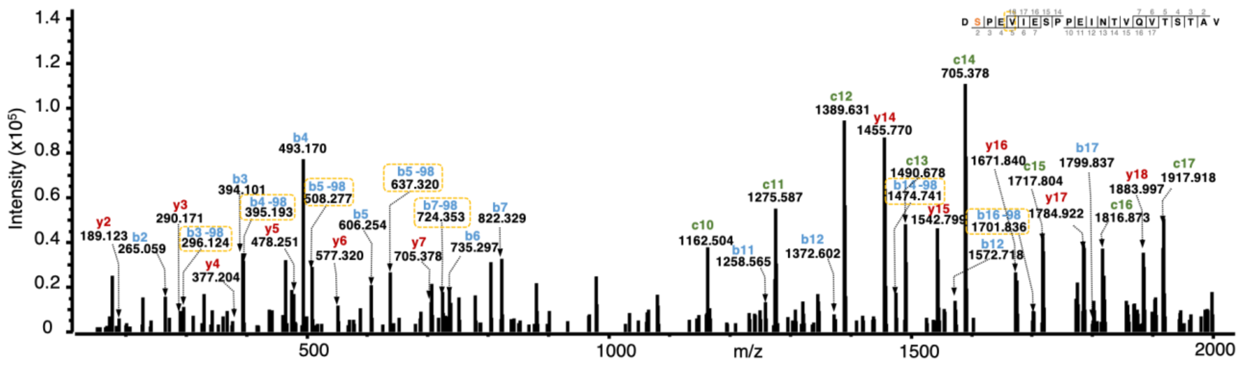

Figure S4. Annotated MS/MS spectra for aCMP fragment with sequence A with phosphorylation. Tandem MS spectra for DSPEVIESPPEINTVQVTSTAV (m/z 2276.082, z=2) had a phosphorylation site at Ser170 found in CMP powder 1 sample.

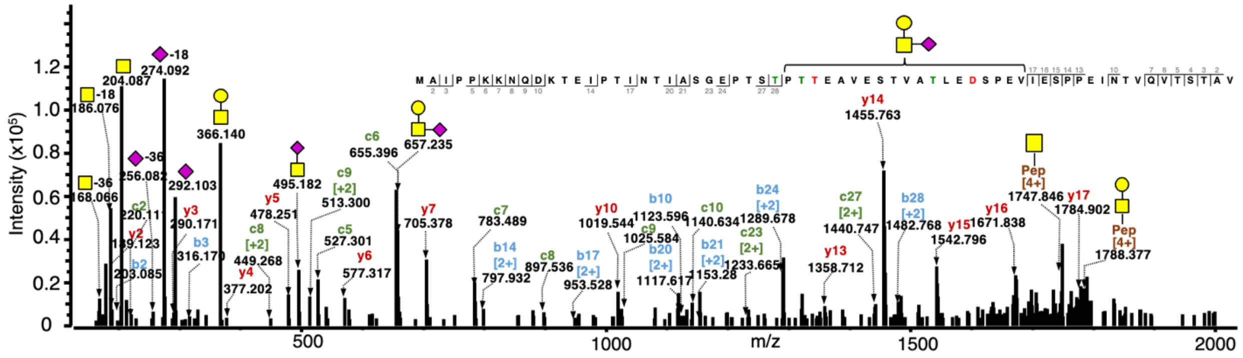

Figure S5. Annotated MS/MS spectra for intact gCMP with sequence A containing trisaccharide O-glycan. Tandem MS spectra for the intact gCMP with sequence B containing one O-glycan (GalNAcGalNeuAc) and one phosphate, observed m/z 1488.924 [M+5H]5+, scan time = 21.68 min found in the CMP standard sample. Glycan symbols: yellow square, N-acetyl galactosamine; yellow circle, galactose; and purple diamond, N-acetyl neuraminic acid.

Table S1. The 51 intact CMPs found in the four samples with peptide sequence, number of phosphorylations, number of oxidations, number of O-glycosylations, glycan structure, observed mass, calculated mass and error ppm.

*Excel S-1

Table S2. The 159 CMP fragments found in the four samples with peptide sequence, number of phosphorylations, number of oxidations, number of O-glycosylations, glycan structure observed mass, calculated mass and error ppm.

*Excel S-2

Table S3. The counts of intact gCMP containing 1, 2 and 3 O-glycans identified in the four samples (CMP STD, CMP powder 1, CMP powder 2 and WPI).

	CMP STD	CMP powder 1	CMP powder 2	WPI
1 *O*-glycan	17	18	18	18
2 *O*-glycans	14	15	16	15
3 *O*-glycans	9	8	9	9
